# Photonic Crystal Polymeric Thin-Film Dye-Lasers for Attachable Strain Sensors

**DOI:** 10.3390/s21165331

**Published:** 2021-08-06

**Authors:** Tsan-Wen Lu, Yu-Kai Feng, Huan-Yeuh Chu, Po-Tsung Lee

**Affiliations:** 1 Department of Photonics, College of Electrical and Computer Engineering, National Yang Ming Chiao Tung University, Rm. 401 CPT Building, 1001 Ta-Hsueh Road, Hsinchu 300093, Taiwan; x135791@gmail.com (Y.-K.F.); chuhuenyuan.ee07@nycu.edu.tw (H.-Y.C.); potsung@nctu.edu.tw (P.-T.L.); 2 Department of Photonics, College of Electrical and Computer Engineering, National Chiao Tung University, Rm. 401 CPT Building, 1001 Ta-Hsueh Road, Hsinchu 300093, Taiwan

**Keywords:** photonic crystal, nanocavity, dye-lasers, strain sensors

## Abstract

In this report, using two-dimensional photonic crystals (PhC) and a one-dimensional PhC nano-beam cavity, we realized the development of all-polymeric dye-lasers on a dye-doped, suspended poly-methylmethacrylate film with a wavelength-scale thickness. In addition to the characterization of basic lasing properties, we also evaluated its capacity to serve as an attachable strain sensor. Through experimentation, we confirmed the stable lasing performances of the dye-laser attaching on a rough surface. Moreover, we also theoretically studied the wavelength responses of the utilized PhC resonators to stretching strain and further improved them via the concept of strain shaping. The attachability and high strain sensing response of the presented thin film PhC dye-lasers demonstrate their potential as attachable strain sensors.

## 1. Introduction

Chemical dyes are one of the mature gain mediums for realizing laser devices. Their light emissions, which cover the near-ultraviolet to near-infrared range, come from the four-level transitions of electrons in dye molecules. Various dyes have been used for realizing fluidic or solid dye-lasers [1,2,3] by dissolving in organic solvents or doping in polymers. Compared with the fluidic dye-lasers, solid dye-lasers [4,5,6,7,8,9,10,11,12,13,14] show more integration compatibilities with different optical platforms and photonic chips. To reduce the threshold and minimize the device footprint of this kind of dye-laser, the dye-doped polymer usually combines with the optical cavities [5,6,7,8,9,10,11] based on different materials. Alternatively, one can directly shape the dye-doped polymer into different types of resonators by a one-step manufacturing process [12,13,14,15,16]. In recent years, researchers have reported solid dye-lasers utilizing various micro and nano-resonators, including, for example, micro-rings [9,11], waveguides [10], micro-disks [12,13,14], and micro-droplets [16] with total internal reflection confinements, or photonic crystal (PhC, including traditional distributed feedback Bragg mirrors) resonators [5,6,15] based on the photonic band and bandgap effects. Recently, researchers further realized plasmonic dye-lasers [7,8] by combining the dye-doped polymer with metallic nanostructures with surface plasmonic resonances.

In addition to studying different laser properties [3,7,8,14], optical sensing applications of solid-dye lasers are also attractive. In recent reports, based on the change of volume or refractive index, the solid dye-lasers have shown their capabilities in monitoring refractive index [4,6], temperature [9], displacement [14,15], biological and physiological parameters [5,11], and so on. However, most of the above sensors are demonstrated in the bulk form or on different optical structures, which limit the flexibility for sensing on different platforms or targets. In this report, we design and manufacture different PhC resonators for realizing dye-lasers on a suspended dye-doped polymer thin film with a wavelength scale. In addition to basic lasing properties, we also confirm the attachment abilities of thin-film PhC dye-lasers and their stable lasing performances after attaching to a rough surface. To initially investigate the potential of the thin-film PhC dye-lasers presented herein in serving as an attachable strain sensor, we also theoretically study and optimize their sensing responses to strain by the concept of strain shaping.

## 2. Manufacturing Photonic Crystal Resonators on Polymer Thin-Film

**Figure 1:**
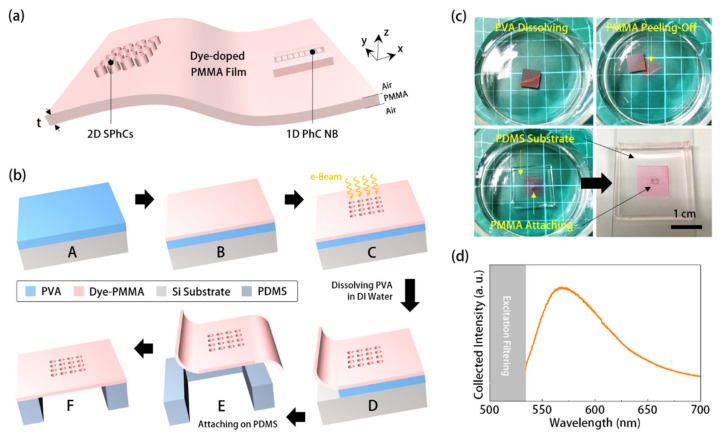
(**a**) Schematics of PhC resonators on a dye-doped PMMA thin film suspending in the air and (**b**) their manufacturing flowchart; (**c**) pictures of separating the dye-doped PMMA thin film from silicon substrate in DI water and attachment on a PDMS template; and (**d**) measured PL spectrum of dye-doped PMMA thin film suspended in the air.

Figure 1a shows schematics of the thin-film PhC dye-laser we studied in this report. It includes two kinds of PhC resonators manufactured on a polymeric thin film (with a thickness of *t*) suspended in the air. The thin film, with a refractive index *n* of 1.49, is doped with chemical dyes as the gain medium. The PhC resonator design includes two-dimensional (2D) square PhCs (SPhCs) and one-dimensional (1D) PhC nanobeam (NB) cavity. In realizing the above structure, the manufacturing process in Figure 1b started with (Step *A*) spin-coating a polyvinyl-alcohol (PVA) interlayer (1000 rpm for 60 s, followed by 3000 rpm for 60 s) on a silicon substrate. We prepared the dye-doped polymer by dissolving the chemical dye PM-597 (Exciton Inc.) in poly-methylmethacrylate (PMMA, 950 K) with a concentration of 25 μmol/g. Afterward, we (Step *B*) spin-coated the dye-doped PMMA onto the PVA layer (1000 rpm for 10 s, followed by 3500 rpm for 25 s), which produced a PMMA layer with a thickness of 300 nm. After soft baking at 90 °C for 180 s, we (Step *C*) directly defined and manufactured the PhC patterns on the dye-doped PMMA layer by electron beam (*e*-beam) lithography. Then, in Steps *D* and *E*, we immersed the sample in deionized (DI) water to dissolve the PVA interlayer and separate the patterned PMMA thin film from the silicon substrate. Finally, we (Step *F*) attached the PMMA thin film onto a polydimethylsiloxane (PDMS) substrate with a via-hole to form a thin film structure suspending in the air. This step also means that one can attach this patterned PMMA thin film onto any other suitable surface or objects. The pictures in Figure 1c show the process from Steps *D* to *F*. We then excited the dye-doped PMMA thin film using a 532 nm laser at room temperature to confirm its optical emission. The measured photoluminescence (PL) spectrum in Figure 1d shows a broad emission spectral linewidth of about 80 nm centered at 570 nm. Therefore, the thickness of the PMMA thin film (*t* = 300 nm) was between *λ*/2*n* and *λ*/*n*, where *λ* represents the emission peak of dye-doped PMMA thin film. This thickness guaranteed that the PMMA thin film, as a planar waveguide in our proposed structure, only supports a transverse-electric-like (*TE*-like) fundamental mode.

## 3. Mode Analysis and Lasing Actions from 1D and 2D PhC Resonators

**Figure 2:**
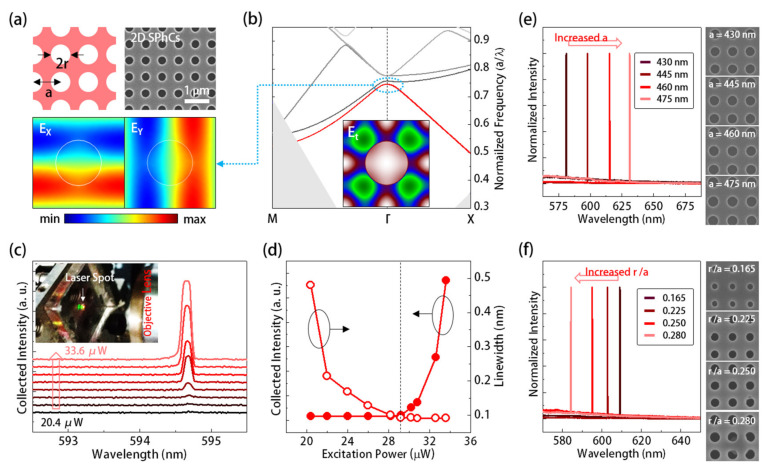
(**a**) Lattice parameters definitions of 2D SPhCs, theoretical *E_X_* and *E_Y_* field distributions of the *Γ_1_* BE mode using 3D FEM simulation, and top-view SEM picture of 2D SPhCs manufactured by the process in Figure 1b; (**b**) theoretical band diagram of 2D SPhCs with *r*/*a* of 0.25 on a dye-doped PMMA thin film with a *t* of 300 nm; the inset shows the theoretical *E_t_* field of *Γ_1_* BE mode by the PWE method for mode identification; (**c**) the measured spectra and (**d**) the corresponding lasing emissions/linewidths under different excitation powers; the inset of (**c**) further shows the picture of light emission from the dye-doped PMMA film with 2D SPhCs; and the measured lasing spectra and SEM images of 2D SPhCs with different (**e**) *a* and (**f**) *r*/*a*.

For the first PhC resonator design, schematics in Figure 2a define the lattice parameters of the 2D SPhCs, including lattice constant *a* and radius *r*. For the 2D SPhCs with an *a* and *r*/*a* of 430 nm and 0.25 on the PMMA thin film with *t* = 300 nm, we calculated its *TE*-like band diagram using the three-dimensional (3D) plane-wave-expansion (PWE) method, as shown in Figure 2b. In Figure 2b, our interested mode candidate is the band-edge (BE) of the dielectric band (the first band, red dispersion curve) near the *Γ* point, denoted as *Γ_1_* BE mode. Its frequency (*a*/*λ* ~ 0.75) aligns with the emission peak of PM-597 dye in Figure 1d. In Figure 2a,b, the theoretical *E_X_*, *E_Y_*, and *E_t_* fields of *Γ_1_* BE mode by 3D finite-element method (FEM, COMSOL Multiphysics software package) and PWE simulations all highly concentrate within the dielectric region. These field concentrations guarantee the strong light-matter interactions between the gain medium and *Γ_1_* BE mode. The flat band near the band-edge standing for low group velocity can also enhance the light-matter interaction in temporal. Furthermore, the BE mode near *Γ* point also means the almost-zero in-plane wave vector can produce vertical radiations when serving as lasers.

Figure 2a shows the scanning electron microscope (SEM) image of the 2D SPhCs on a dye-doped PMMA, manufactured by the process in Figure 1b, which is within an area of 50*a* × 50*a*. We excited it by using a 532 nm laser pulse with a pulse width of 0.5 ns, a repetition rate of 1 kHz, and a spot size of 8 μm in diameter, at room temperature. Figure 2c and its inset show the measured spectra under excitation powers from 20.4 to 33.6 μW and the picture of light emission (the orange light spot) from the dye-doped PMMA film with 2D SPhCs under excitation. Figure 2d further shows the collected power (light-in-light-out (*L-L*) curve) and spectral linewidth variations of the optical emissions of the 2D SPhCs under different excitation powers. The curves’ bending clearly indicate the lasing action with a threshold of 29 μW. To prove this lasing action comes from the *Γ_1_* BE mode, instead of the dye itself, we further characterized the emissions from 2D SPhCs with different lattice parameters. In Figure 2e, for the 2D SPhCs with a fixed *r*/*a* of 0.26 and increased *a* from 430 to 475 nm, the lasing wavelengths showed significant red shifting. Furthermore, in Figure 2f, with a fixed *a* of 445 nm, the lasing wavelengths of 2D SPhCs showed a blue shifting when the *r*/*a* increased from 0.165 to 0.28. These wavelength shifts, caused by different lattice parameters, are the features of photonic modes in PhCs. It thus confirms the laser emissions come from the *Γ_1_* BE mode in 2D SPhCs.

**Figure 3:**
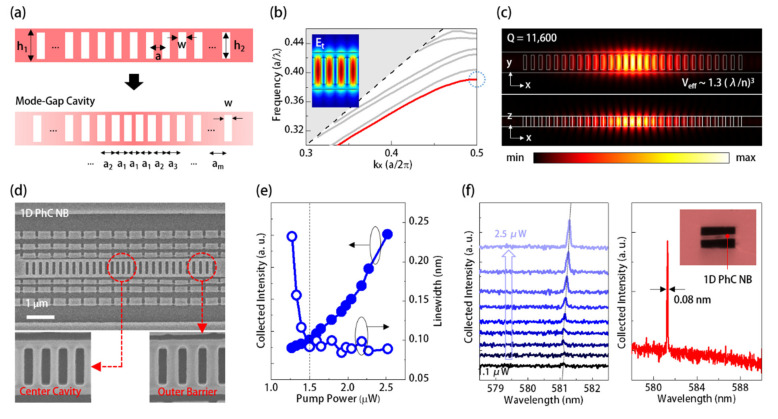
(**a**) Schematics and lattice parameter definitions of 1D PhC NB on a dye-doped PMMA thin film and the nanocavity formed by linearly increased lattice constants; (**b**) theoretical *TE*-like band diagram of 1D PhC NB with *w*, *h_1_*, *h_2_*, *a*, and *t* of 110, 700, 500, 220, and 300 nm; (**c**) theoretical *E_t_* distributions of the dielectric mode confined in the 1D PhC NB cavity along *XY*- and *XZ*-planes; (**d**) top- and zoom-in view SEM images of 1D PhC NB cavity manufactured by the process in Figure 1b; (**e**) the measured laser emissions/linewidths and (**f**) spectra of 1D PhC dye-laser under different excitation powers; and the inset of (**f**) also shows the optical microscope (OM) image of the 1D PhC NB cavity.

In the second kind of PhC resonator design, the 1D PhC NB consisted of periodic rectangular holes with height *h_2_* and width *w* arranged along a suspended dye-doped PMMA waveguide with width *h_1_*, as defined in Figure 3a. In Figure 3b, the theoretical *TE*-like band diagram of the 1D PhC NB by the PWE method showed that the first band was a dielectric band, which is beneficial for realizing nanolasers. To effectively locally confine this dielectric band, we linearly increased the lattice constant of PhCs by 5 nm from the center to the edges of the NB to form a nanocavity, as illustrated in Figure 3a. Although PMMA produces low index contrast (0.49) relative to the air when guiding the optical wave in the PhC NB, the above double hetero-lattice design provided a mode-gap effect [17] for locally confining and tailoring the dielectric mode. Theoretical *E_t_* field distributions of the confined dielectric mode along *XY*- and *XZ*-planes in Figure 3c clearly show its field concentration within the nanocavity region. In the case of the 15 holes on each side of the nanocavity (i.e., the *m* defined in Figure 3a is 14), the confined dielectric mode is with a sufficiently high-quality factor (*Q*) of 11,600 and a small effective mode volume (*V_eff_*) of 1.26 (*λ*/*n*)^3^. This high *Q*/*V_eff_* value is beneficial for providing strong light-matter interactions as a mode candidate in a nanolaser.

Figure 3d shows the top and zoom-in view SEM pictures of the manufactured 1D PhC NB cavity on a dye-doped PMMA thin film before separating from the silicon substrate. By the same excitation condition for 2D SPhCs, Figure 3e shows the *L-L* curve and spectral linewidths under different excitation powers from 1.5 to 2.5 μW, where the curves’ bending indicate a low lasing threshold of 1.5 μW. Figure 3f shows the corresponding lasing spectra, which show a narrow spectral lasing linewidth of 0.08 nm. It should be noted that the threshold of the dye-laser based on the 1D PhC NB cavity is one order lower than that of the *Γ_1_* BE mode in the 2D SPhCs in Figure 2d. This low threshold comes from the strong light-matter interactions provided by the confined dielectric mode with a high *Q*/*V_eff_* value and the reduced redundant gain medium by a small device footprint of the 1D PhC NB cavity. Although the *Γ_1_* BE mode in the 2D SPhCs has a much higher threshold, its strong emissions, standing for a high signal-to-noise (*S/N*) ratio, is still beneficial for optical sensing.

In addition, owing to the high excitation laser pulse repetition rate (1 kHz), all the activated PhC dye-lasers will be at least exposed to over 5 × 10^5^ excitation pulses during a power-dependence PL measurement. However, we did not observe significant intensity degradation or wavelength blue shift caused by the bleaching of the dye, as shown in Figure 2c,d and Figure 3e,f. Therefore, we believe the dye-lasers presented could handle tens of minutes to several hours of accumulated operation time, under a lower excitation repetition rate of several tens Hz, which is long enough for executing sensing applications.

## 4. Evaluating on Feasibility as an Attachable Strain Sensor

**Figure 4:**
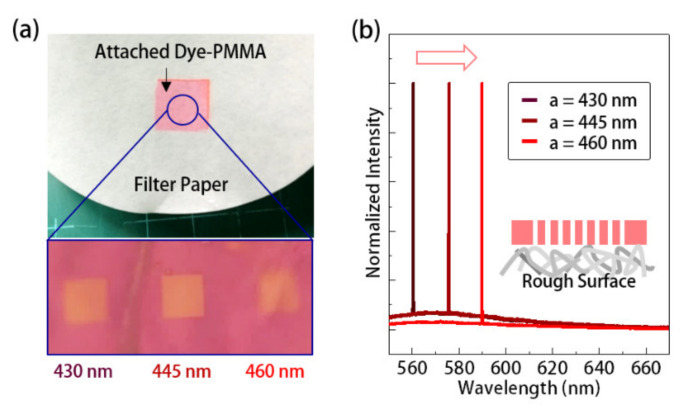
(**a**) Picture and OM image of 2D SPhCs thin-film dye-lasers with different lattice constants on a filter paper and (**b**) their lasing spectra.

In addition to a low-cost visible laser, the single-mode lasing action and narrow spectral linewidth are also beneficial for providing a sufficient *S/N* ratio and spectral resolution in optical sensing applications. Owing to the elastic thin film structure, the PhC dye-lasers presented herein could further be used as a strain sensor, capable of on-demand attachment to analyzed object surfaces. To confirm the feasibility of this idea, we first investigated the lasing properties of the PhC dye-lasers after attaching them to an object’s surface. In the manufacturing process, we separated the dye-doped PMMA thin film from the silicon substrate and attached it to filter paper in DI water. The picture and OM image in Figure 4a show the dye-doped PMMA thin film with the 2D SPhCs with different lattice constants on filter paper. In measurements, the attached 2D SPhC dye-lasers retain almost the same lasing wavelength in the case of suspending it in the air, as shown in Figure 4b. These invariant wavelengths come from attaching to a surface with roughness much larger than the wavelength scale, which makes the 2D SPhCs equivalent to suspension in the air, as illustrated by the inset of Figure 4b. The lasing wavelength also showed the same trend of increasing with the lattice constant. Therefore, we confirmed our presented PhC dye-lasers were still workable when attached to the analyzed object surface, particularly to a surface with a roughness larger than *λ*. In addition, it of note that this thin film with PhC dye-lasers can be transferred to the other object’s surface by merely repeating the above-attaching process in water, which means it has reusability when serving as an attachable strain sensor.

**Figure 5:**
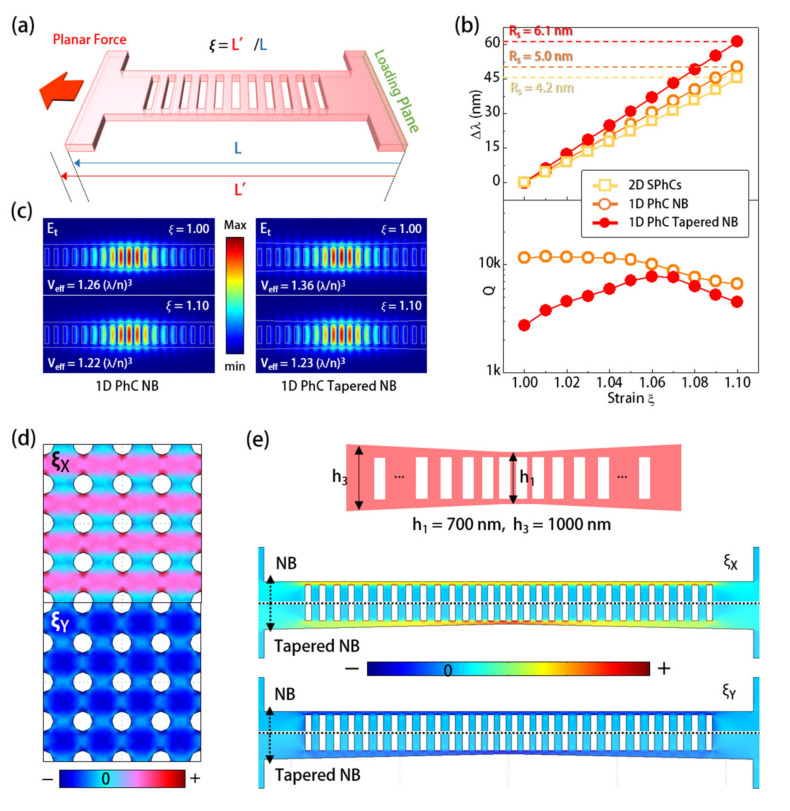
(**a**) Schematics of stretching sample in FEM simulation and definition of stretching strain *ξ*; (**b**) under different applied strain *ξ* along the *X*-direction, theoretical (top) wavelength shifts and (bottom) *Q* of the dielectric and *Γ_1_* BE modes in 1D PhC untapered/tapered NB cavities and 2D SPhCs; (**c**) theoretical *E_t_* fields of the dielectric modes in 1D PhC untapered and tapered NB cavities under applied stretching strain *ξ* = 1.00 and 1.10 along *X*-direction; (**d**) theoretical *ξ_X_* and *ξ_Y_* distributions of 2D SPhCs under an applied stretching strain along the *X*-direction; and (**e**) design of 1D PhC cavity with tapered NB width and the theoretical *ξ_X_* and *ξ_Y_* distributions of tapered and untapered NB cavities under an applied strain along the *X*-direction.

To theoretically investigate the capabilities of strain sensing of the 2D SPhCs and 1D PhC NB cavity, we applied a stretching strain *ξ* to them along the *X*-direction and characterized their optical responses by the FEM simulation setup in Figure 5a. In Figure 5a, we set a loading plane on one side of the PMMA thin film with PhC resonators and applied a planar force on the opposite side. The applied stretching strain ξ was defined as the length ratio of the sample before (*L*) and after (*L*′) applying stretching. Figure 5b shows the wavelength shifts of these two resonator designs under every 1% *ξ* variation (defined as strain response *R_S_*) under the applied *ξ* from 1.00 to 1.10, which correspond to an *R_S_* of 4.2 and 5.0 nm, respectively. In evaluating the strain sensing capability, the expression *Δλ*/*R_S_* defined the minimum detectable strain variation *Δξ_det_*, where *Δλ* represents the spectral linewidth of the utilized mode decided by *λ/Q* in a passive PhC resonator. However, in an active resonator, *Δλ* represents the lasing spectral linewidth, which is 0.08 nm as shown in Figure 2c and Figure 3e, while the *Q* value mainly determines the lasing threshold level. Therefore, the *R_S_* values of the utilized modes, depending on PhC lattice structures and device topologies, will determine the sensing performance. In addition to sensing performance, the utilized mode stability in PhC dye-lasers under the applied strain is also important for the strain sensor. For example, Figure 5b shows theoretical *Q* values of dielectric mode in the 1D PhC NB cavity under *ξ* from 1.00 to 1.10, where *Q* is almost invariant under *ξ* < 1.05 and only slightly degraded with an enlarged *ξ* under *ξ* > 1.05. In Figure 5c, the theoretical *E_t_* fields and *V_eff_* of the dielectric mode in the 1D PhC NB cavity under *ξ* of 1.00 and 1.10 were also almost invariant. These properties mean the dielectric mode can be well maintained inside the nanocavity under the applied *ξ*, which thus guarantees stable laser properties during strain sensing.

Figure 5d,e shows the theoretical strain distributions, along the *X*- (ξ_X_) and *Y*-directions (*ξ_Y_*) of the above PhC resonator designs, under the applied stretching strain *ξ* along the X-direction, which can explain their difference in *R_S_* value. In the case of 2D SPhCs, although the resulted *ξ_X_* stretched the lattice along the *X*-direction, the resultant *ξ_Y_* compressed the lattice instead along the *Y*-direction because of the positive Poisson ratio (~0.33) of PMMA, as shown in Figure 5d. That means the responses of *Γ_1_* BE mode to the applied strain will be different in different lattice directions, which thus limits its *R_S_* value. On the contrary, in the case of the 1D PhC NB, because the lattices are arranged along only one direction, the *ξ_X_* in Figure 5e thus becomes the only shaper to the lattices and dielectric mode inside, while *ξ_Y_* only slightly compresses the NB along the *Y*-direction without significantly deforming the lattices. Therefore, the dielectric mode in the 1D PhC NB cavity had a higher *R_S_* of 5.0 nm.

To further enhance the *R_S_* of the dielectric mode in the 1D PhC NB, we modified the NB to have a tapered width by *h_1_* to *h_3_*, as shown in Figure 5e. This non-uniform NB design, based on the concept of strain shaping [18,19,20], can concentrate more strain in the narrow region (center of NB) under uniformly applied stress. In Figure 5e, the comparison between theoretical *ξ_X_* of the 1D PhC tapered and untapered NB cavities clearly confirmed this kind of strain concentration. Even the *ξ_Y_* for slightly compressing the NB was also minimized in this tapered NB design, as shown in Figure 5e. Therefore, via the maximized *ξ_X_*, this design can produce an improved *R_S_* value of 6.1 nm, as shown in Figure 5b. In addition, in Figure 5b,c, the theoretical *Q* and *V_eff_* of the dielectric mode in this tapered NB cavity also showed small variations under different applied *ξ*, which can guarantee stable laser properties during strain sensing. As a strain sensor, according to the expression *Δλ*/*R_S_*, with a lasing spectral linewidth *Δλ* of 0.08 nm in Figure 3f and an *R_S_* of 6.1 nm, the theoretical *Δξ_det_* of the dielectric mode in the 1D PhC tapered NB cavity could be 1.3 × 10^−4^, which is smaller than that (~2.0 × 10^−4^) provided by the *Γ_1_* BE mode in the 2D SPhCs. Moreover, according to the *ξ_X_* and *ξ_Y_* distributions in Figure 5e, it is reasonable to assume that we could further enhance the *R_s_* value of the dielectric mode in the tapered NB cavity by enlarging the ratio between the NB side walls widths near and away from the cavity region, i.e., enlarging the value of (*h_3_*-*h_2_*)/(*h_1_*-*h_2_*), to concentrate more strain near the cavity region. However, it would also accompany *Q* degradation, as shown in Figure 5b.

On the other hand, because of the high thermo-optic coefficient of PMMA used herein, our proposed PhC dye-lasers would also be suitable for temperature sensing. In evaluating the sensing response, the wavelength shifts *λ_T_* (in a unit of nm/K) of the dielectric mode in the 1D PhC NB cavity, caused by temperature variation, can be expressed as:

*λ_T_* = α × *R_v_* + (*dn*/*dT*) × *R_i_* (1)

where α is the coefficient of thermal expansion in a unit of K^−1^, which represents the material expansion with temperature. *R_v_* represents the dielectric mode wavelength shift 1% isotropic expansion of the PhC NB. We should note that this *R_v_* is different from the *R_s_* obtained from the NB under an anisotropic expansion by the positive Poisson ratio. The α × *R_v_* term thus represents the dielectric mode wavelength shift caused by the PhC NB expansion with temperature. In the (*dn*/*dT*) × *R_i_* term, the thermo-optic coefficient *dn*/*dT* represents the PhC NB refractive index change with temperature, while *R_i_* represents the dielectric mode wavelength shift caused by the PhC NB refractive index change. Therefore, the (*dn*/*dT*) × *R_i_* term represents the dielectric mode wavelength shift caused by the PhC NB refractive index change with temperature. For the PMMA (950 K) used in this report, α and *dn*/*dT* were approximately 4.2 × 10^−5^ K^−1^ and −3.2 × 10^−4^ K^−1^, respectively. The theoretical *R_v_* and *R_i_* of the dielectric mode in the 1D PhC tapered NB cavity were 5.8 and 289 nm, respectively. According to Equation (1), the theoretical *λ_T_* of the dielectric mode can be determined as −0.068 nm/K. Considering the minimum linewidth (0.08 nm) of the NB laser presented herein, its minimum detectable temperature variation *ΔT_det_* would be 1.2 K when serving as a temperature sensor. For the *Γ_1_* BE mode with *R_v_* and *R_i_* of 5.9 and 220 nm, its theoretical *λ_T_* of −0.046 nm/K would result in a *ΔT_det_* of 1.7 K.

## 5. Conclusions

In this report, by *Γ_1_* BE mode in the 2D SPhCs and confined dielectric mode in the 1D PhC NB cavity, we realized dye-lasers on a suspended dye-doped PMMA thin film with a wavelength-scale thickness. In measurement, in addition to directional laser emissions and a low lasing threshold of 1.5 μW, the single-mode lasing with narrow spectral linewidth were also beneficial for providing a sufficient *S/N* ratio and spectral resolution in spectrometry optical sensing applications. Moreover, owing to the elastic thin film structure, the PhC dye-lasers presented herein could further be used as a strain sensor capable of on-demand attachment to analyzed object surfaces. To show the feasibility of this idea, we first confirmed the stable lasing performance of the 2D PhC dye-lasers after attaching them to a filter paper. Afterward, we theoretically analyzed the sensing responses of the *Γ_1_* BE and dielectric modes to the stretching strain via mechanical and electromagnetic simulations. Moreover, to further improve the sensing response of dielectric mode in the 1D PhC NB cavity, we proposed a novel design of a 1D PhC cavity with a tapered NB width based on the concept of strain shaping. This design allowed for more strain concentration in the nanocavity region and thus produced an improved *R_S_* value of 6.1 nm and minimum detectable strain variation *Δξ_det_* as low as 1.3 × 10^−4^. Therefore, we believe that our proposed thin film PhC dye-lasers would be feasible and have potential for use as an attachable strain sensor.
